# Finding HSP neighbors via an exact, hierarchical approach

**DOI:** 10.1016/j.is.2025.102565

**Published:** 2025-05-21

**Authors:** Cole Foster, Edgar Chávez, Benjamin Kimia

**Affiliations:** a Brown University, Providence, RI, USA; b Centro de Investigación Científica y de Educación Superior de Ensenada, Mexico

**Keywords:** Similarity search, Half-Space Proximal graph, RNG, Exact indexing, Hierarchical indexing

## Abstract

The Half Space Proximal (HSP) graph is a low out-degree monotonic graph with a wide range of applications in various domains, including combinatorial optimization in strings, enhancing kNN classification, simplifying chemical networks, estimating local intrinsic dimensionality, and generating uniform samples from skewed distributions, among others. However, the linear complexity of finding HSP neighbors of a query limits its scalability, thus motivating approximate indexing which sacrifices accuracy in favor of restricting the test to a small local neighborhood. This compromise leads to the loss of crucial long-range connections which as a result introduce false positives and exclude false negatives, and compromising some of the essential properties of the HSP. To overcome these limitations, this paper proposes a fast and exact algorithm for computing the HSP which enjoys sublinear complexity as demonstrated by extensive experimentation. Our hierarchical approach leverages the triangle inequality applied to pivots to enable efficient HSP search in metric spaces with the Hilbert Exclusion property. A key component of our approach is the concept of the *shifted generalized hyperplane* between two points, which allows for the invalidation of entire groups of points. Our approach ensures the computation of the exact HSP with efficiency, even for datasets containing hundreds of millions of points.

## 1. Introduction

The topology of a dataset — the structure of local and global relationships between elements — is fundamental to efficient similarity search. Proximity graphs, such as the Relative Neighborhood Graph (RNG) and the Half-Space Proximal (HSP) graph, encode this topology by connecting points that satisfy geometric criteria, enabling navigation strategies using this knowledge. The HSP graph, in particular, offers a sparse, directed, and *monotonic* structure, where paths between nodes guarantee non-increasing distance to the target. This property makes the HSP indispensable for applications like routing in ad-hoc networks, median string computation, instance-based classification, and local intrinsic dimensionality estimation. However, a critical barrier persists: *the computational cost of finding exact HSP neighbors remains prohibitive for large datasets*.

Existing methods to compute the HSP graph rely on the *HSP Test*, an iterative process that prunes candidate neighbors using half-space exclusion criteria. While the per-query complexity of this test is linear in the dataset size N, constructing the full HSP graph requires applying it to all N elements, resulting in $O\left(N^{2}\right)$ time. To mitigate this, state-of-the-art implementations restrict the HSP Test to a small local neighborhood around the query, approximating the graph by only considering candidates within a fixed radius or among approximate k-nearest neighbors. While this reduces complexity, it discards *long-range HSP links* — connections that span distant regions of the dataset — which are vital to preserving the HSP’s monotonicity and global structural integrity. For example, Fig. 3 demonstrates how approximate methods fail to retain critical links that ensure connectivity between clusters, degrading downstream performance in tasks like classification or density estimation.

This compromise raises a fundamental challenge: *how to compute the exact HSP graph efficiently without sacrificing its defining properties*. Exact methods are not merely theoretical ideals; they are essential for applications where approximation errors propagate catastrophically, such as distance oracles, spanner networks, and intrinsic dimensionality estimation. Yet, the linear per-query complexity of brute-force HSP Tests limits scalability, and hierarchical acceleration structures used in metric-space search (e.g., cover trees, M-trees) are not directly applicable due to the HSP’s unique dependency on iterative half-space exclusion.

**Fig. 1.**
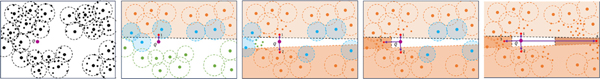
A visualization of the sequence for the proposed Hierarchical HSP algorithm, where pivots are used to invalidate entire groups of points without having to calculate distances to query or testing inequalities.

In this work, we address this challenge by introducing a hierarchical, exact algorithm for HSP neighbor retrieval that achieves sublinear search complexity while preserving 100% recall. Our approach relies on pivot-based hierarchies and the *shifted generalized hyperplane* criterion to invalidate or retain entire groups of points during the iterative HSP Test, avoiding costly pairwise distance computations. By organizing the dataset into multi-resolution pivot domains, we exploit the triangle inequality and metric-space geometry to bound distances and exclude irrelevant regions, noticeably reducing the number of candidates evaluated, Fig. 1. Experiments on datasets with up to 13 million points demonstrate that our method reduces search time by up to 348x compared to brute force, outperforms approximate HSP variants in high-recall regimes, and scales efficiently across dimensions — all while exactly preserving the HSP’s monotonic property.

## 2. Related work

Computing the exact k-nearest neighbor (kNN) graph in intrinsically high-dimensional spaces incurs a quadratic $O\left(N^{2}\right)$ time complexity, as it requires calculating pairwise distances between all N points. This becomes computationally prohibitive for large datasets, as distances in high-dimensional spaces lack meaningful contrast (the “curse of dimensionality”), rendering space-partitioning acceleration structures ineffective. These methods, *e.g.*, the classical kd-trees [1] and subsequent refinements, which rely on geometric properties to prune search regions, degrade to brute-force linear scans in high dimensions due to the loss of spatial locality.

State-of-the-art approaches for large-scale or high-dimensional data universally resort to approximate methods. Techniques like Hierarchical Navigable Small World (HNSW) graphs [2], Locality-Sensitive Hashing (LSH) [3], and more general graph-based approximations prioritize sublinear query times by trading exactness for efficiency. For instance, HNSW constructs layered proximity graphs that enable logarithmictime neighbor retrieval, while LSH uses hash functions to probabilistically group nearby points. These methods exploit heuristics or probabilistic guarantees to bypass exhaustive distance comparisons, reducing complexity to O(NlogN) or better. However, they introduce trade-offs in recall or distance accuracy, as true nearest neighbors may be missed.

Approximations are unavoidable in practice due to the quadratic bottleneck of exact methods, which becomes insurmountable for modern datasets. Even optimized parallel implementations or distributed computing frameworks cannot circumvent the asymptotic scaling. Additionally, many applications — such as clustering, classification, or recommendation systems — tolerate minor inaccuracies in neighbor selection, making approximations a pragmatic compromise. Thus, while exact kNN graphs remain a theoretical ideal, approximations dominate real-world use cases, balancing computational feasibility with sufficient accuracy for downstream tasks.

On the other hand, proximity graphs such as the Relative Neighborhood Graph (RNG), the Gabriel Graph, *etc.*, use triplets of elements at a time to determine the occupancy of a neighborhood region, *e.g.*, a lune defined by two elements, by a third element. This gives a richer sense of topology to a neighborhood.

While kNN graphs and other pairwise-distance graphs can unevenly distribute neighbors, possibly clustered in one direction, proximity graphs embed a sense of direction in their neighborhood definition, albeit requiring the additional cost associated with considering triplets of elements. Analogously, the Yao graph [4] and the Theta graph [5] construct a sense of direction by partitioning the space around each point into equal-angle cones. For the Yao graph, each point $x_{i}$ divides the space into k cones with apex at $x_{i}$, and connects to the nearest neighbor within each cone, ensuring neighbors are spread across directions. This cone-based strategy reduces computational complexity compared to proximity graphs like the RNG, as it avoids exhaustive triplet checks. However, the Yao graph’s reliance on angular partitioning inherently ties it to Euclidean spaces, where angles and directional cones are well-defined.

The Theta graph, a related structure, refines this idea. For instance, the Θ-5 graph [5] partitions the plane into six 60° cones and connects each point to the “closest” point in each cone, where closeness is measured by the projection onto the cone’s bisector rather than Euclidean distance. This projective criterion ensures the graph is a spanner — a subgraph that approximately preserves shortest paths — with a bounded stretch factor. While both Yao and Theta graphs enforce directional diversity, the Theta graph’s edge selection rules often yield sparser yet more navigable structures.

Crucially, these methods are confined to Euclidean spaces due to their dependence on angular geometry and vector projections. In contrast, the HSP graph operates in general metric spaces, circumventing such geometric constraints. Furthermore, while Yao/Theta graphs avoid the $O\left(N^{3}\right)$ complexity of RNG construction, they still require O(NlogN) preprocessing for cone partitioning and nearest-neighbor searches within cones. This trade-off-lower computational cost at the expense of geometric flexibility—highlights the distinct advantages of the HSP approach in non-Euclidean domains.

The Half-Space Proximal (HSP) graph [6] aims to capture a sense of direction, but without the computational requirements of proximity graphs, specifically the RNG, and without the Yao/Theta graphs’ restrictions to a Euclidean space. Like an RNG, it connects two elements if the lune between them is empty, but unlike the RNG which checks occupancy by *all* other elements, HSP checks occupancy only by those elements already established as closer HSP neighbors. This recursive definition requires a treatment of elements rank-ordered by distance to the query.

Formally, consider a dataset S of elements $\left\{x_{1},x_{2},\ldots ,x_{N}\right\}$ and a query point Q. The RNG lune between two elements, say $x_{i}$ and $x_{j}$, is the intersection of the two “spheres” of radius $d\left(x_{i},x_{j}\right)$ centered at $x_{i}$ and $x_{j}$, *i.e.*, the set of x satisfying both conditions

(1a) $$\left\{\begin{array}{l} d\left(x,x_{i}\right)<d\left(x_{i},x_{j}\right)\\ d\left(x,x_{j}\right)<d\left(x_{i},x_{j}\right),\;\text{(1b)} \end{array}\right.$$

**Fig. 2.**
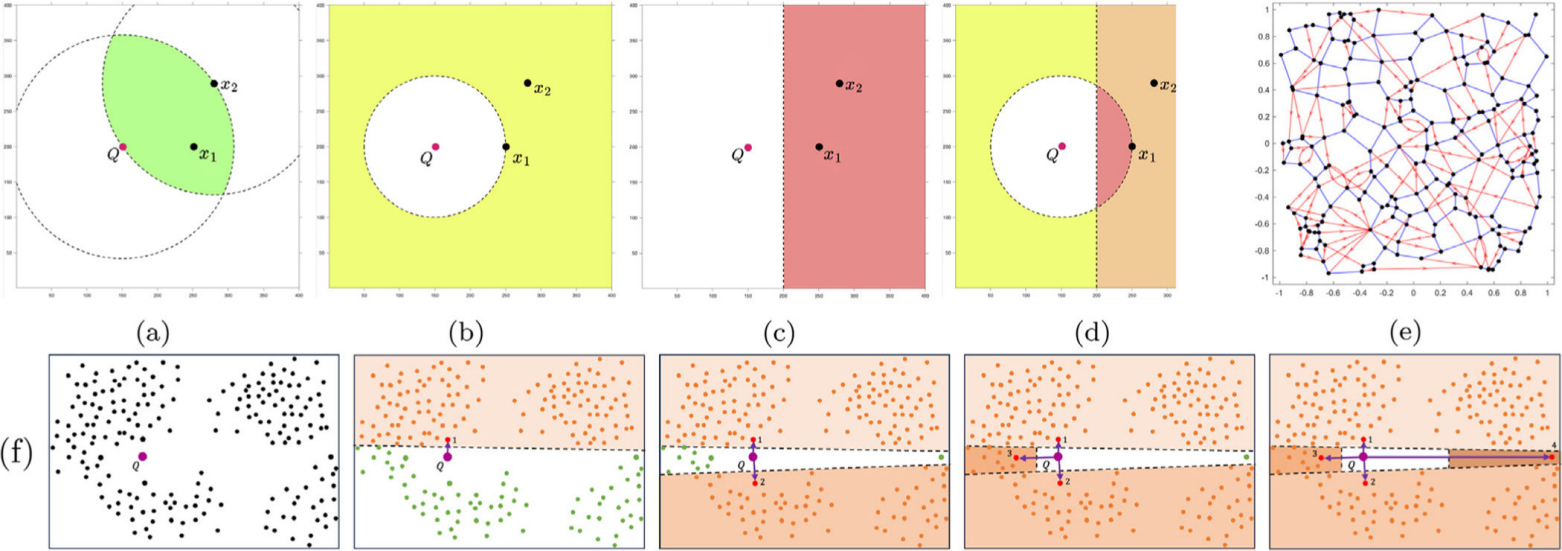
(a) An RNG link between Q and $x_{2}$ is conditional on an empty RNG lune (green), and the presence of $x_{1}$ in the RNG lune between Q and $x_{2}$ disrupts this link. The region of $x_{2}$ whose link with Q is invalidated is the intersection of the region corresponding to Eq. (3)(a) shown in (b) and the region corresponding to Eq. (3)(b) shown in (c), with the intersection shown in (d). The RNG and HSP Graph on a 2D dataset of uniformly distributed points with the RNG links are shown in blue and the additional, directed HSP links are shown in red. (f) A visualization of the sequence for finding the HSP neighbors of Q among the points shown on the left. Each of the nearest neighbors rules out a half-space (shaded area). (For interpretation of the references to color in this figure legend, the reader is referred to the web version of this article.)

Fig. 2(a). The RNG graph is defined by the set of links between pairs of elements $\left(x_{i},x_{j}\right)$ for which all other elements $x_{k}\in S$ fall outside their lune. This process requires a consideration of all triplets, an $O\left(N^{3}\right)$ process. The HSP graph is similar to the RNG in constructing the lune condition, but it aims to tame the computational expense by limiting the consideration of which $x_{k}$ occupy the RNG lune. Specifically, observe that in considering links with a particular element Q, the nearest neighbor to Q, say $x_{1}$, has an RNG link with Q since the first of the two required conditions,

(2a) $$\left\{\begin{array}{l} d\left(x_{k},Q\right)<d\left(Q,x_{1}\right)\\ d\left(x_{k},x_{1}\right)<d\left(Q,x_{1}\right),\;\text{(2b)} \end{array}\right.$$

cannot be met by any $x_{k}$ by construction. In the case that two elements or more are equidistant to Q, they are all RNG neighbors like $x_{1}$. Now, consider another element $x_{2}$ not equal to $x_{1}$ or any other equidistant neighbors, and ask when is it that $x_{2}$ cannot have an RNG link with Q because $x_{1}$ is in its lune, *i.e.*,

(3a) $$\left\{\begin{array}{l} d\left(x_{1},Q\right)<d\left(Q,x_{2}\right)\\ d\left(x_{1},x_{2}\right)<d\left(Q,x_{2}\right)?\;\text{(3b)} \end{array}\right.$$

Clearly, the first condition is always met, *i.e.*, $x_{2}$ is outside the circle of radius $d\left(Q,x_{1}\right)$ centered at Q, Fig. 2(b), the region shown in yellow. The second condition represents the set of $x_{2}$ on the right *half-space* shown as red in Fig. 2(c). The intersection of the two regions represents the set of all $x_{2}$ which *cannot* form an RNG link with Q because of the presence of $x_{1}$, Fig. 2(d), and this entire region, effectively the half-space, can be eliminated as a RNG neighbor of Q. Next, the nearest neighbor to Q on the remaining half-space, say $x_{3}$, has a potential RNG link with Q. This potential link might be ruled out as an RNG link due to the presence of some $x_{2}$ that was eliminated. However, disregarding this possibility gives rise to the notion of the HSP graph: Q links to $x_{3}$ with an HSP link. This second HSP link in turn rules out another half-space. This process is then repeated with the nearest neighbor in the remaining space resulting in the HSP Graph when all elements are considered, Fig. 2(e). Observe that RNG⊂HSP. The process is simulated in Fig. 2(f).

### 2.1. Efficient approaches to computing the HSP

**Fig. 3.**
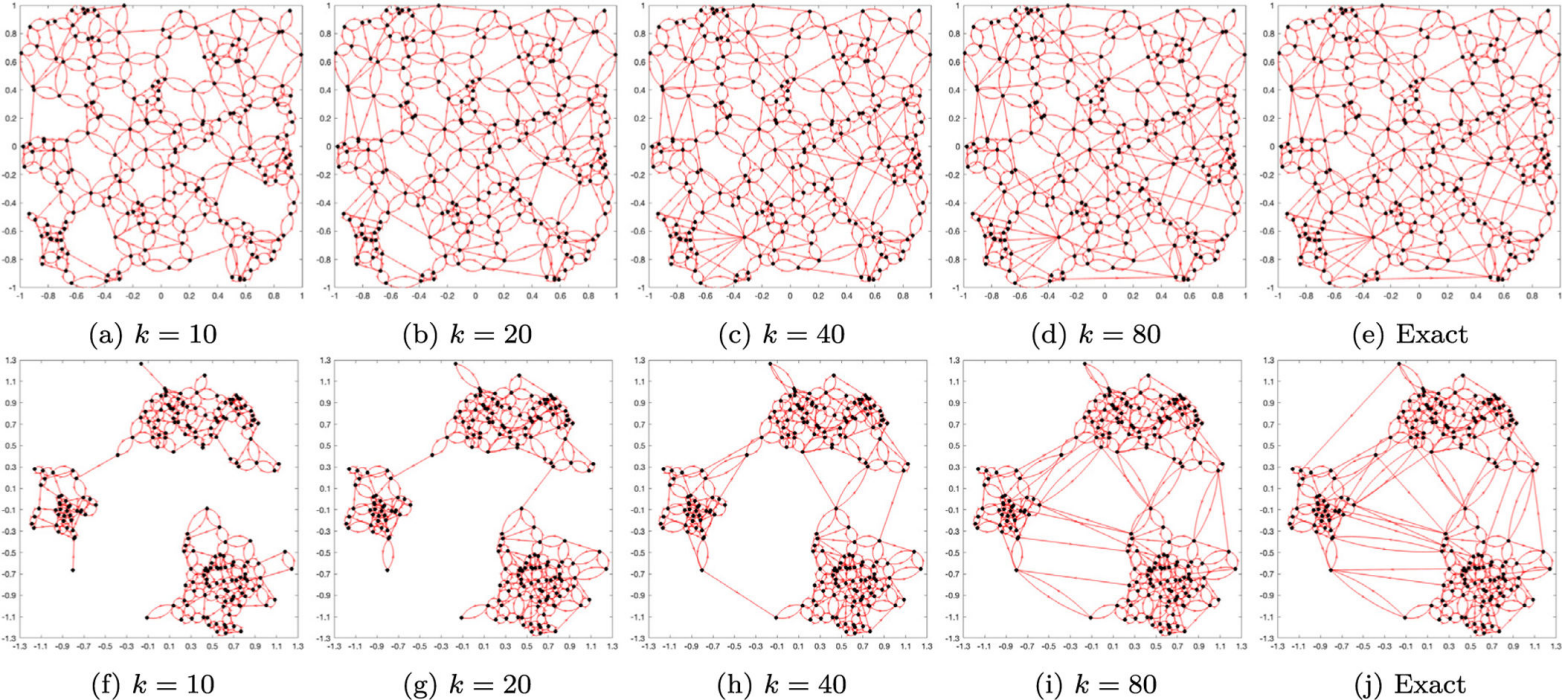
A dataset of N=200 uniformly distributed 2D points (top row) and a dataset of N=200 clustered points (bottom row) are used to compare the exact HSP Graph (e,j), to the approximate HSP Graph where the HSP neighbor candidates are restricted to the k closest points, for k=10,20,40,80.

The HSP neighbors of a query point Q are defined algorithmically, through the iterative process described above. This algorithm was first introduced as the *HSP Test* [6], used as a “test” to prune edges in ad-hoc networks. The HSP Graph [7] is defined by independently applying the HSP Test to each individual node, which enables a distributed graph construction. Each iteration of the HSP Test requires an exact nearest neighbor search to find the next HSP neighbor and the application of Eq. (3) to validate each member of the dataset, both of which are O(N) processes on the first iteration and results in an overall O(N) process for all iterations due to the rapid decline in the number of points considered. The HSP Graph requires applying the HSP Test to all N elements independently, giving an overall construction complexity of $O\left(N^{2}\right)$, which can be daunting for large-scale datasets. Yet, despite the numerous benefits and applications of the HSP graph (see below), there has been surprisingly few attempts at reducing the complexity of finding the HSP neighbors of a query, the key component in constructing the HSP graph, and of value in its own right. All existing approaches restrict the HSP neighbors to a local area around Q: in the original paper [6] that introduced the HSP graph, the HSP neighbors are constrained to the points within a small distance of Q. More recently, in the application of instance-based classification [8], the HSP neighbors were restricted to the approximate kNN (k=300) of Q using HNSW [2]. However, any method of restricting the HSP Test to some local area will inevitably result in an approximation. Consider Fig. 2(f), where the fourth HSP neighbor lies very far away from Q. These types of links are often those non-RNG neighbors that are a result of near-parallel generalized hyperplanes invalidating nearby points and thus preventing them from invalidating further ones. Fig. 3 shows how approximate methods miss these long range links which are significant as they preserve some of the essential qualities of the HSP Graph, including its monotonic property described next.

### 2.2. HSP is a monotonic graph

A fully monotonic graph is a graph where each pair of nodes (u,v) lies on a *monotonic path* $u=u_{0},u_{1},u_{2},\ldots ,u_{\ell +1}=v$ where $d\left(u_{i+1},v\right)\leq d\left(u_{i},v\right)$ for all i. The notion emerged in 1985 when Dearholt [9] proposed the Monotonic Search Network (MSNET), a graph constructed by adding edges to the RNG, as the ideal graph for “search without backtracking”. Although monotonicity does not guarantee exact nearest neighbor search for queries outside the dataset, it is useful in defining theoretical guarantees of accuracy and search path length [10,11]. The prohibitive quadratic construction complexity of monotonic graphs have led a variety of approximate versions. Some approaches simply ensure a monotonic path between a single starting node and all members of the dataset to preserve the theoretical guarantees [10,12], but a single starting point becomes more susceptible to local minima in practice. A common approach to graph construction for similarity search is to start with some initial graph, such as a kNN graph [10] or an empty graph [2], and perform an approximate kNN search on each node to incrementally update the edges of each node. To improve the sparsity of the retrieved neighbors while preserving diversity, the HSP Test is often used to prune the edges, essentially defining the neighbors of each node as its approximate HSP neighbors [2,10,13]. This edge selection strategy locally preserves monotonicity, but will fail to capture the long-range links that are vital to the monotonic property of the HSP Graph, Fig. 3.

## 3. Applications of HSP

The initial application of the HSP graph was for routing between nodes in *ad-hoc networks* where the challenge is using only local information without central control. Another application is in the challenging optimization problem of finding the “median string” in a dataset, *i.e.*, the string with minimum distance to all other strings [14]. The median string has various applications in different domains, such as bioinformatics, pattern recognition, and data compression. In bioinformatics, the median string can be used for DNA and protein sequence analysis, where it helps in identifying consensus sequences and motifs. In pattern recognition, the median string can be used as a representative prototype for a set of strings, enabling efficient nearest-neighbor classification and clustering, *e.g.*, for handwritten character recognition. In data compression, the median string can be used as a reference for encoding a set of similar strings, as it minimizes the total encoding cost. By representing the strings in terms of their differences from the median, the data can be compressed more efficiently. Additionally, the median string finds applications in error correction, where it can help in correcting errors in strings by comparing them to the median of a set of valid strings.

The HSP neighbor finding has been used to enhance the majority-rule neighborhood classifiers where the kNN neighbors are replaced with the HSP neighbors, eliminating the need to set the parameter k, which is challenging to determine. The candidate neighbors come from a probabilistic index such as the HNSW [2].

Another application of the HSP graph is in representing chemical networks [15,16]. The typically used complete graph or an α-similarity graph are significantly reduced in size by retaining only HSP neighbors, reducing quadratic memory requirements to linear ones.

The HSP neighbors have also been used in local intrinsic dimensionality estimation [17]. The connection between the maximum degree of the HSP graph and the kissing or sphere packing number (the maximum number of mutually touching spheres in a Euclidean space) is used to define the indexability of a set.

In [18], the authors define the *hubness* HSP (HubHSP) graph, a graph which preserves the geometric structure of the HSP neighbors but favors neighbors as nodes with highest “hubness”, or centrality, which can be used to sample skewed distributions, *e.g.*, for pivot selection.

### 3.1. The computation of exact HSP is significant

Finding exact HSP neighbors is key to a number of problems including determining the local intrinsic dimension as well as local density estimation. Also, a standing conjecture about the HSP is about it being t-spanner of finite stretch t. This has a potential application as a distance oracle. Storing the entire distance matrix of a metric database implies $O\left(N^{2}\right)$ space of precomputed distances, while storing the HSP graph uses space proportional to O(N×δ(HSP(S))), where δ(.) is the degree of the graph. Using the monotonic property of the HSP the oracle can be consulted in time proportional to the diameter of the graph. For the above oracle to work, it is mandatory to compute the HSP exactly to obtain a proper bound.

### 3.2. Hierarchies in exact metric-space similarity search

A key approach to addressing the computational complexity of computing exact HSP neighbors is to use *pivots*, select points in the dataset. The triangle inequality is used to bound the distance between the query point and other members of the dataset, removing a large portion from consideration. For example, the List of Clusters (LOC) [19] organizes the dataset into an ordered list of pivots, where each pivot is responsible for a group of points within a radius of that pivot. The query can traverse the list, only considering the clusters that may contain the nearest neighbor. Observing that an increase an N also increases the number of points in each cluster, the Recursive List of Clusters [20] improves efficiency by organizing each cluster into its own LOC. In fact, this recursive organization of the dataset into smaller and smaller groups is the basic concept of tree structures, which achieve logarithmic search complexity. Some metric-space tree structures of interest include the M-Tree [21], the Cover Tree [22], and several others [23–25].

### 3.3. Proposed method overview

The proposed approach constructs a multilayer hierarchy of coarse-scale pivots “owning” finer-scale pivots in their pivot domains defined by a radius. The hierarchy is then used to (*i*) find the nearest neighbors to a query without computing distance to each element by relying on conditions on pivots and (*ii*) use pivot conditions to discard entire domains of elements from consideration of being HSP neighbors, preserve entire domains as active, or declare domains as indeterminant which require examination of its members. The approach preserves the iterative form of the HSP algorithm, making each iteration more efficient, and the sequence is depicted in Fig. 1. This results in significant savings and leads to scalable HSP computation on large-scale, high-dimensional datasets featuring clustered data. Our implementation is publicly available at https://github.com/Brown-LEMS/HHSP.git.

## 4. Hierarchical retrieval of exact HSP neighbors (HHSP)

The main bottleneck in finding the HSP neighbors of a query is (*i*) the computation of the distance to *all* dataset members, and (*ii*) to a lesser extent, the validation of the inequalities in Eq. (3). The key intuition is to use a hierarchy where pivots representing a group of dataset points can be examined and used to either discard or retain from consideration the entire elements in the pivot domain, thus avoiding explicit computation of distances or validation of inequalities.

Specifically, recall that HSP neighbors are found by discarding half-spaces. Denote the elements that are not discarded as the active list A. First, the distance between Q and all dataset members is calculated to return the closest point to Q, $x_{1}$, which becomes the first HSP neighbor; $x_{1}$ is used to discard a half-space from being HSP neighbors of Q by checking Eq. (3) on the remaining dataset members. Next, the closest point among the active list A which is the set of surviving dataset members becomes the second HSP neighbor and is used to check and discard the remainder of the active list that satisfy Eq. (3). This process is repeated until all members are labeled either as HSP neighbors or discarded, Fig. 2(f).

**Fig. 4.**
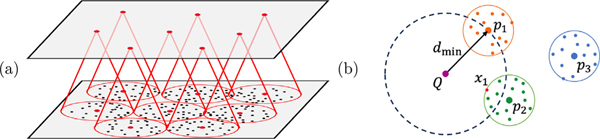
(a) A two-layer hierarchy where the bottom layer contains all of S and the top layer has a select few pivots where the distance of dataset elements to its pivot parent is less than r. (b) Given an upper-bound distance $d_{\min}$, a pivot p may contain the nearest neighbor $x_{1}$ only if $d(Q,p)\leq d_{\min}+r$. (For interpretation of the references to color in this figure legend, the reader is referred to the web version of this article.)

A first key savings can be achieved through the computation of the nearest neighbor of Q by using pivots in a hierarchy. Consider, at first, a two-layer hierarchy where the “bottom” layer is the dataset and the “top” layer is a select group of elements of the dataset called pivots $p_{i}$ such that each data point $x_{j}$ is in the domain of one, and only one, pivot $p_{i}$, namely, $d\left(x_{j},p_{i}\right)\leq r$, where r is a fixed parameter of the hierarchy, Fig. 4(a). There are numerous ways to construct this hierarchy. The approach used here is to randomly consider the dataset members one by one and assign them to either belonging to an existing pivot or if no pivot can be found, assign it as a pivot. The parameter r determines the size of the pivot domain.

The pivot structure can be used to significantly reduce the computational effort in finding the nearest neighbor of Q. Let $d_{\min}$ denote the minimum distance of the query Q to the data elements already considered. Then, the distance from Q to any other element x in the pivot domain of p satisfies the triangle inequality

(4) d(Q,p)−r≤d(Q,p)−d(p,x)≤d(Q,x)≤d(Q,p)+d(p,x)≤d(Q,p)+r.

Thus, if for a pivot p, $d(Q,p)>d_{\min}+r$, then

(5) $$d_{\min}<d(Q,p)-r\leq d(Q,x),$$

and the pivot domain cannot contain the nearest element, see the blue pivot domain associated with $p_{3}$ in Fig. 4(b). On the other hand, if $d(Q,p)\leq d_{\min}+r$, the green pivot domain in Fig. 4(b), the elements in the domain of p must be explicitly considered and if one has a lower distance than $d_{\min}$, it updates $d_{\min}$. This prevents the computation of distances to a vast majority of dataset elements. The process is repeated until all pivots have been considered in this way. A better performance can be achieved by using tighter bounds if each pivot would maintain the distance to its most distant member, $r^{*}$.

A second key savings can be achieved through wholesale examination of Eq. (3) for all members of a pivot domain *without* calculating distance to query or validation of inequalities by examining d(Q,p). The following proposition prevents member-wise validation of the second inequality in Eq. (3) if the pivot satisfies certain conditions.

### Proposition 1.

*Let* Q *be a query*, $x_{1}$ *the furthest HSP neighbor of* Q *thus far*, *and* $p_{2}$ *a pivot with domain radius* r *satisfying the following:*

(6) $$d^{2}\left(Q,p_{2}\right)-d^{2}\left(x_{1},p_{2}\right)>2r\;d\left(Q,x_{1}\right)$$

*Then*, *all points* $x_{2}\in D\left(p_{2},r\right)$, *i.e.*, *where* $d\left(x_{2},p_{2}\right)\leq r$, *satisfy* $d\left(x_{1},x_{2}\right)<d\left(Q,x_{2}\right)$.

**Proof.** By the assumption of a metric space satisfying the Hilbert Exclusion principle [26] ensures that all points $x_{2}$ satisfying $d\left(x_{2},p_{2}\right)\leq r$ further satisfy $d\left(x_{1},x_{2}\right)<d\left(Q,x_{2}\right)$ when Eq. (6) holds. ■

A geometric interpretation of Eq. (6) in Euclidean space gives an intriguing perspective that the locus of $p_{2}$ is the half-space to the right of a shifted generalized hyperplane in a Euclidean space, because the quadratic terms involving coordinates of $p_{2}$ cancel out leaving a linear equation which represents a hyperplane. Returning now the first inequality in Eq. (1), the following proposition identifies the condition on a pivot so that the entire pivot domain can be discarded.

### Proposition 2.

*Let* Q *be a query and* $x_{1}$ *the furthest HSP neighbor of* Q *thus far*, *then a pivot* $p_{2}$ *with radius* r *satisfying both of the following inequalities*

(7a) $$\left\{\begin{array}{l} d^{2}\left(Q,p_{2}\right)-d^{2}\left(x_{1},p_{2}\right)>2r\;d\left(Q,x_{1}\right)\\ d\left(Q,x_{1}\right)<d\left(Q,p_{2}\right)-r,\;\text{(7b)} \end{array}\right.$$

*invalidates all points* $x_{2}\in D\left(p_{2},r\right)$, *i.e.*, $d\left(p_{2},x_{2}\right)\leq r$, *as a HSP neighbors of* Q.

**Proof.** First, let us show that $d\left(Q,x_{1}\right)<d\left(Q,x_{2}\right)$:

(8) $$\begin{array}{l} d\left(Q,x_{1}\right)<d\left(Q,p_{2}\right)-r\leq d\left(Q,x_{2}\right)+d\left(x_{2},p_{2}\right)-r\leq d\left(Q,x_{2}\right)+r-r\\ \;=d\left(Q,x_{2}\right). \end{array}$$

Second, since $0\leq d\left(Q,x_{1}\right)$, Eq. (7)(b) shows that $d\left(Q,p_{2}\right)>r$ which together with satisfy Proposition 1 which states that $d\left(x_{1},x_{2}\right)<d\left(Q,x_{2}\right)$, the second inequality of Eq. (3) holds. ■

**Fig. 5.**
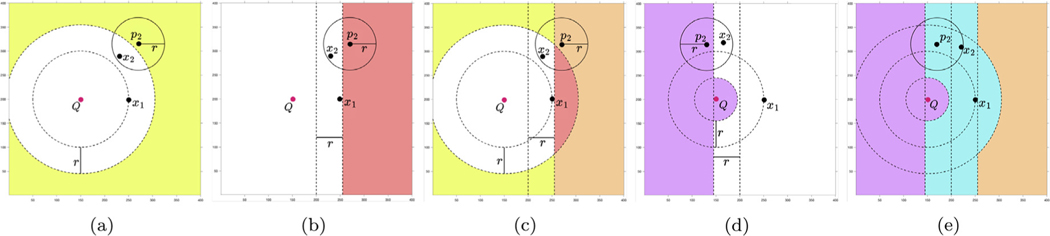
(a) The point $x_{2}$ will satisfy Eq. (3)(a) when its parent pivot $p_{2}$ is outside of the circle of radius $d\left(Q,x_{1}\right)+r$ centered at Q. (b) The point $x_{2}$ satisfies Eq. (3)(b) when $p_{2}$ is to the right of the generalized hyperplane shifted by r. (c) The intersection of the two regions (orange) which defines locations for $p_{2}$ where its pivot domain members cannot be an HSP neighbor of Q. (d) The point $x_{2}$ does not satisfy both inequalities of Eq. (3) when $p_{2}$ falls into the purple region. (e) When $p_{2}$ falls into the blue region, it is undetermined if $x_{2}$ satisfies both inequalities of Eq. (3). (For interpretation of the references to color in this figure legend, the reader is referred to the web version of this article.)

The regions corresponding to Eqs. (7)(b) and Eqs. (7)(a) are shown in Fig. 5(a) and 5(b), respectively, leading to their common intersection in Fig. 5(c).

In addition to determining which pivot domains are entirely ruled out, it is also possible to determine which pivot domains cannot get ruled out in their entirety because their members do not satisfy either the first or the second inequalities in Eq. (3) and can therefore remain on the active list in their entirety.

### Proposition 3.

*Let* Q *be a query*, $x_{1}$ *be the furthest HSP neighbor of* Q *thus far*, *and* $p_{2}$ *a pivot satisfying either of the following:*

(9a) $$\left\{\begin{array}{l} d^{2}\left(x_{1},p_{2}\right)-d^{2}\left(Q,p_{2}\right)\geq 2r\;d\left(Q,x_{1}\right)\\ d\left(Q,p_{2}\right)\leq d\left(Q,x_{1}\right)-r.\;\text{(9b)} \end{array}\right.$$

*Then*, *all points* $x_{2}\in D\left(p_{2},r\right)$, *i.e.*, $d\left(p_{2},x_{2}\right)\leq r$, *violate one of the inequalities of Eq. (3)*, *i.e.*, *either* $d\left(Q,x_{1}\right)\geq d\left(Q,x_{2}\right)$ *or* $d\left(x_{1},x_{2}\right)\geq d\left(Q,x_{2}\right)$.

**Proof.** First, Eq. (9)(b) implies that

(10) $$d\left(Q,x_{2}\right)\leq d\left(Q,p_{2}\right)+d\left(p_{2},x_{2}\right)\leq d\left(Q,p_{2}\right)+r\leq d\left(Q,x_{1}\right).$$

Second, by the Hilbert Exclusion principle, Eqs. (9)(a), and $d\left(p_{2},x_{2}\right)\leq r$, r≥0, it holds that $d\left(x_{1},x_{2}\right)\geq d\left(Q,x_{2}\right)$. ■

Fig. 5(d) visualizes the regions defined by the inequalities in Eqs. (9)(a) and (9)(b), which is the union of the shifted half-space and a reduced radius disk. A pivot $p_{2}$ in the purple region is retained in the active list without detailed examination of its elements.

The pivots that are neither fully discarded (orange area) nor fully accepted as surviving in their entirety (purple area) can potentially contain elements which can be discarded and elements that survive (cyan area), Fig. 5(e). The elements in these pivot domains must be individually tested with the inequalities of Eq. (3). However, this

**Algorithm 1:** Hierarchical HSP Search on a 2-Layer Hierarchy

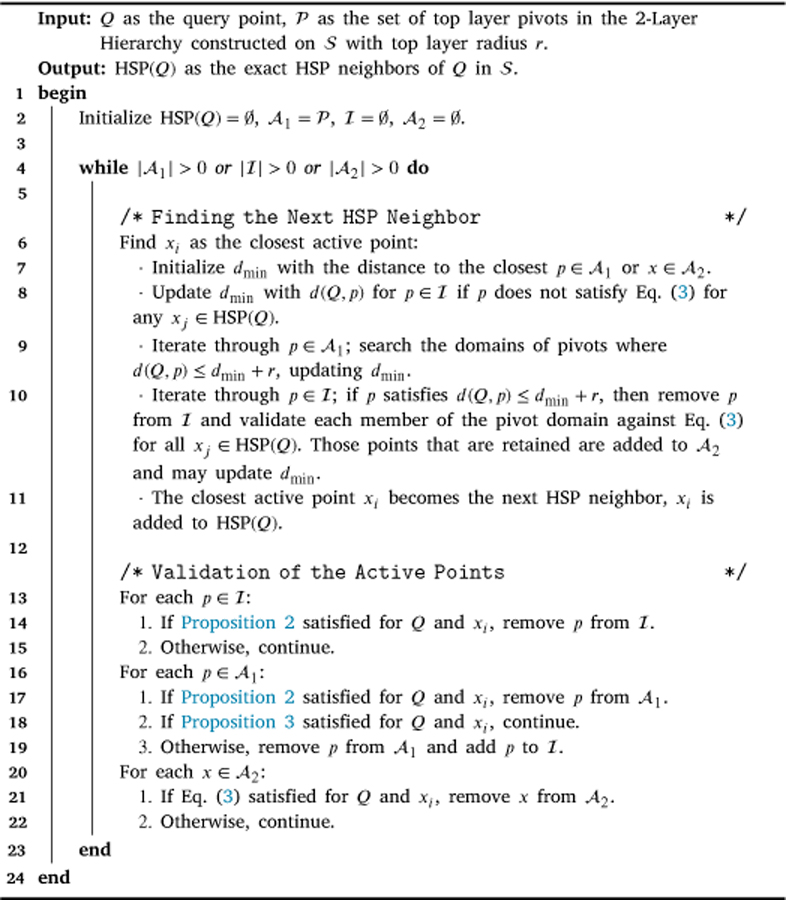

determination can be delayed until the point where their elements need to be examined. It is entirely possible that this entire pivot domain would be discarded in the next steps.

The details of the procedure are in Algorithm 1. Basically, the hierarchy is used to efficiently find the nearest neighbor, Proposition 2 and 3 are used to discard entire pivot domains and retain an active pivot list $A_{1}$ (purple area) of pivots, and an indeterminant list I (cyan area). The procedure is then repeated by finding the next nearest element by exploring pivots in $A_{1}\cup I$. In the process, some of the pivots in I may have to be explicitly examined. The pivots are removed from I, and added to an active point list $A_{2}$ of elements. The process is repeated by finding the nearest element in $A_{1}\cup I\cup A_{2}$ until they are all exhausted.

### 4.1. Multi-layer hierarchies

**Fig. 6.**
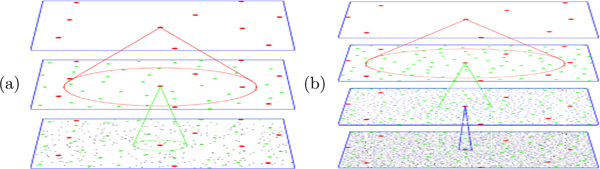
(a) Depiction of a three-layer hierarchy. (b) Depiction of a four-layer hierarchy.

**Fig. 7.**
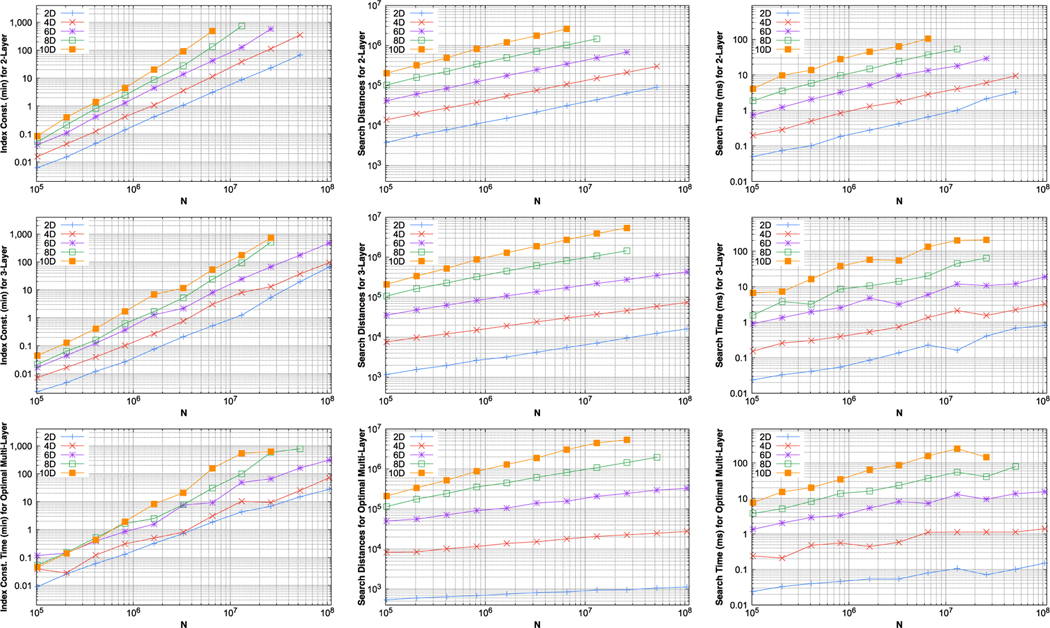
Index construction time, average number of distance computations on search, and average search time for HHSP on 2D-10D data using 2-Layer (top row), 3-Layer (middle row), and optimal multi-layer (bottom row) indices. Observe that these plots are approximately linear in the log–log domain.

This two-layer hierarchical approach achieves efficiency by using pivots to avoid the consideration of a vast number. of points. As N increases, the number of pivots and the number of points in each pivot domain must both increase. This motivates the use of additional layers, similar to other hierarchical indices [21–25]. Just as pivots are able to discard or retain an entire pivot domain of elements, coarse-scale (large radius) pivots discard or retain pivot domains of finer-scale pivots, *e.g.*, Fig. 6. Additional layers can achieve enhanced efficiency when N is increased further, Fig. 7.

### 4.2. The Hilbert exclusion condition

The Hilbert Exclusion condition, proposed in [26], is a stronger condition than the triangle inequality which offers an alternative way to exclude parts of the search space when performing similarity search in metric spaces. By the triangle inequality, any three points in a metric space can be embedded in a 2-dimensional space such that the pairwise distances are preserved. Certain metric spaces are isometrically 4-embeddable in 3-dimensional Euclidean space, meaning that any four points can be embedded in a 3-dimensional space preserving all pairwise distances. This is not true for all metric spaces, meaning that only a subset of metric spaces can take advantage of the Hilbert Exclusion principle, including the popular Euclidean spaces, spaces using Jensen–Shannon distance, spaces with Triangular distance, and some Cosine distance spaces. Metric spaces that do not have this property include spaces using Manhattan distance, Chebyshev distance, Hamming distance, and Levenshtein distance [26]. Despite these limitations, spaces satisfying the Hilbert exclusion condition are relevant to a wide range of applications, including network design and analysis [27], geographic information systems (GIS) [28], signal processing [29], phylogenetics [30], genomics [31,32], and social network analysis [33].

## 5. Experiments

Our experimental evaluation assesses the efficiency, scalability, and accuracy of the Hierarchical HSP (HHSP) across synthetic and real-world datasets. We compare HHSP to brute-force HSP and approximate methods, analyze its performance under varying dimensionality and data distributions, and validate its pivot selection strategies.

All experiments were conducted on a machine with an Intel Xeon E5-2680v4 CPU (2.4 GHz), 256 GB RAM, and Ubuntu 20.04. The HHSP implementation, brute-force HSP, and approximate baselines (HNSW-HSP, kNN-HSP) were written in C++ with OpenMP for parallelism. Results are averaged over 1000 queries. Our key metrics were **Search Time**: Wall-clock time per query (milliseconds). **Distance Computations**: Number of pairwise distance calculations per query. **Recall**: Fraction of exact HSP neighbors retrieved. **Index Construction Time**: Time to build the hierarchical pivot structure (seconds).

### 5.1. Datasets

- **Synthetic Uniform Data**: Generated N points uniformly in ${\left[0,1\right]}^{D}$ for D=2,4,6,8,10 and N up to 13.1 million. Used to measure scalability and dimensionality effects.
- **Synthetic Clustered Data**: Created by sampling 100 cluster centers uniformly in ${\left[-1,1\right]}^{D}$, then generating 1000 points per cluster via Gaussian perturbations (σ=0.05). Total N=100,000 points. Evaluates performance on non-uniform, real-like distributions.
- **Real-World Datasets**:
  - **LA**: 1.07 million 2D geographic coordinates of locations in Los Angeles.
  - **Forest**: 580,812 6D points describing forest cover types (elevation, slope, soil type, etc.).
  - **ImageNet Color Histograms**: 1.28 million 32D HSV histograms from ImageNet-1k images.

### 5.2. HHSP complexity on uniformly distributed data

The HHSP’s complexity is examined on uniformly distributed data of varying dimension, Fig. 7. Observe that the indexing construction time, the average number of distances for search, and the average search time all depict an approximately linear profile against N for varying numbers of layers in a log–log domain. A straight line in log–log is $\log(y)=\alpha \;\log(N)+\gamma \;\text{or}\;y=\beta N^{\alpha }$. While theoretical complexity bounds have not been defined, the above experiments suggest an approach to characterizing complexity of the HHSP, Table 1, by reporting the exponent α, where α=1 suggests linear complexity and α=2 suggests quadratic complexity. The search time complexity of the HHSP is highly sub-linear and improves as the number of layers increases, and the index construction time is sub-quadratic and similarly improves with the number of layers.

### 5.3. Comparison to brute force

**Fig. 8.**
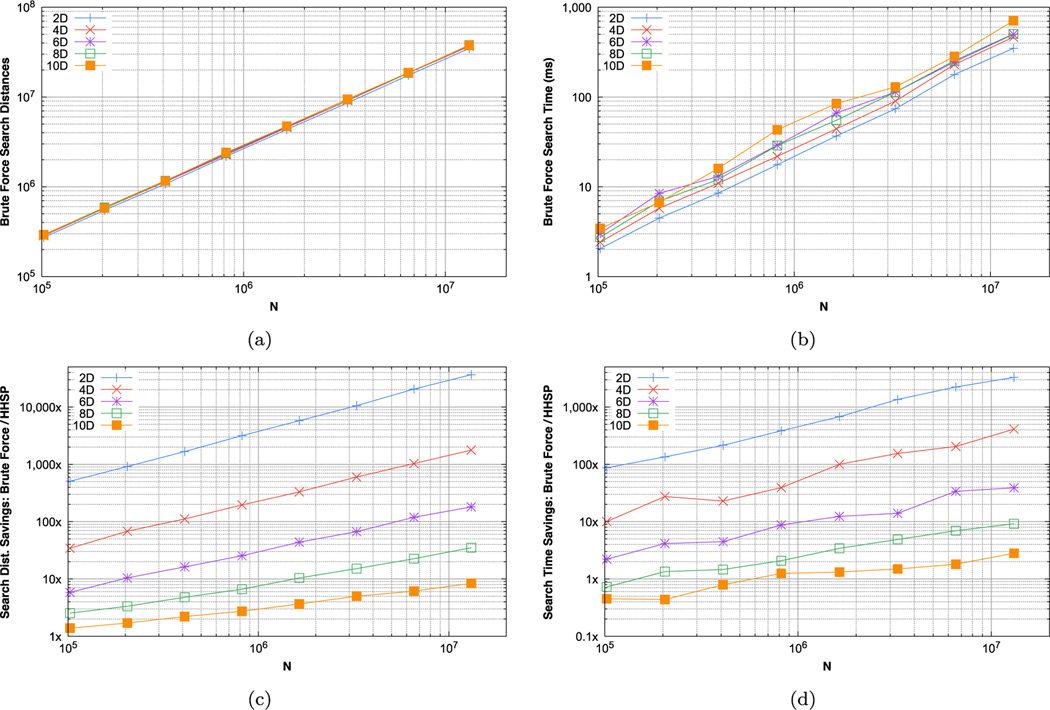
The results of the brute-force HSP algorithm savings of the HHSP over the brute force HSP algorithm in ratios of (a) the average number of search distances per N, (b) the ratio of average distance computations in comparison over those required for the brute force HSP test, and (c) the ratio of the average search time and that required for brute force search. It is evident that the index remains effective and its efficiency increases with N.

The results of the brute force HSP come from a highly optimized implementation, showing the predicted linear complexity scaling to 13 million points within a search latency of 1 s, Fig. 8(a–b). The HHSP significantly outperforms the brute force HSP, Fig. 8(c–d), *e.g.*, the HHSP achieves time savings of 38x in a 6D dataset of N=13,107,200 points. As N increases for a fixed dimension, the savings over brute-force increase, showing that HHSP is effective on high dimensional datasets with sufficient size.

### 5.4. HHSP search on clustered data

**Fig. 9.**
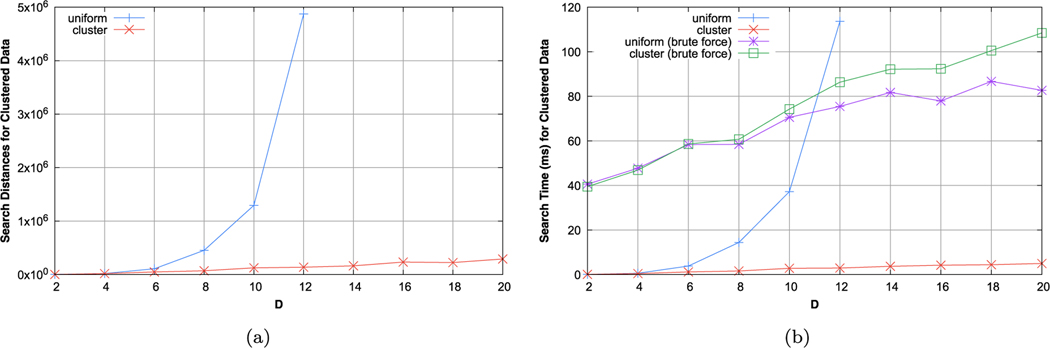
A comparison of HHSP performance on uniformly distributed data vs. clustered data for N=100,000 shows a significantly reduced rate of increase in distance computations (a) and search time (b).

Realistic datasets are typically not uniformly distributed. Rather, data points are often clustered, *e.g.*, object categories. The performance of the HHSP is measured on a synthetic clustered dataset created by initializing 100 uniformly distributed clustered centers from ${\left[-1,1\right]}^{D}$ and using Gaussians with variance = 0.05 to create 1000 perturbations of each center, Fig. 9. Note that the rate of increase with D for the search time and the number of distance computations is significantly lower.

### 5.5. Real world datasets

The performance of HHSP on real world datasets, Table 2. (1) *LA*, a 2D dataset of the geographic locations in Los Angeles with N=1,073,827 points; (2) *Forest*, a 6D dataset of quantitative variables used for classifying forest cover types; and (3) *ImageNet Color Histograms*, a collection of 32D HSV color histograms captured from the N=1,281,167 images of the ImageNet-1k training set [34], which was inspired by the Corel dataset [35]. The HHSP significantly outperforms the brute force HSP Test for exact HSP search, leading to 348.5x, 86.2x, and 2.8x savings over brute force time in LA, Forest, and ImageNet, respectively.

### 5.6. Comparison to approximate HSP search

**Fig. 10.**
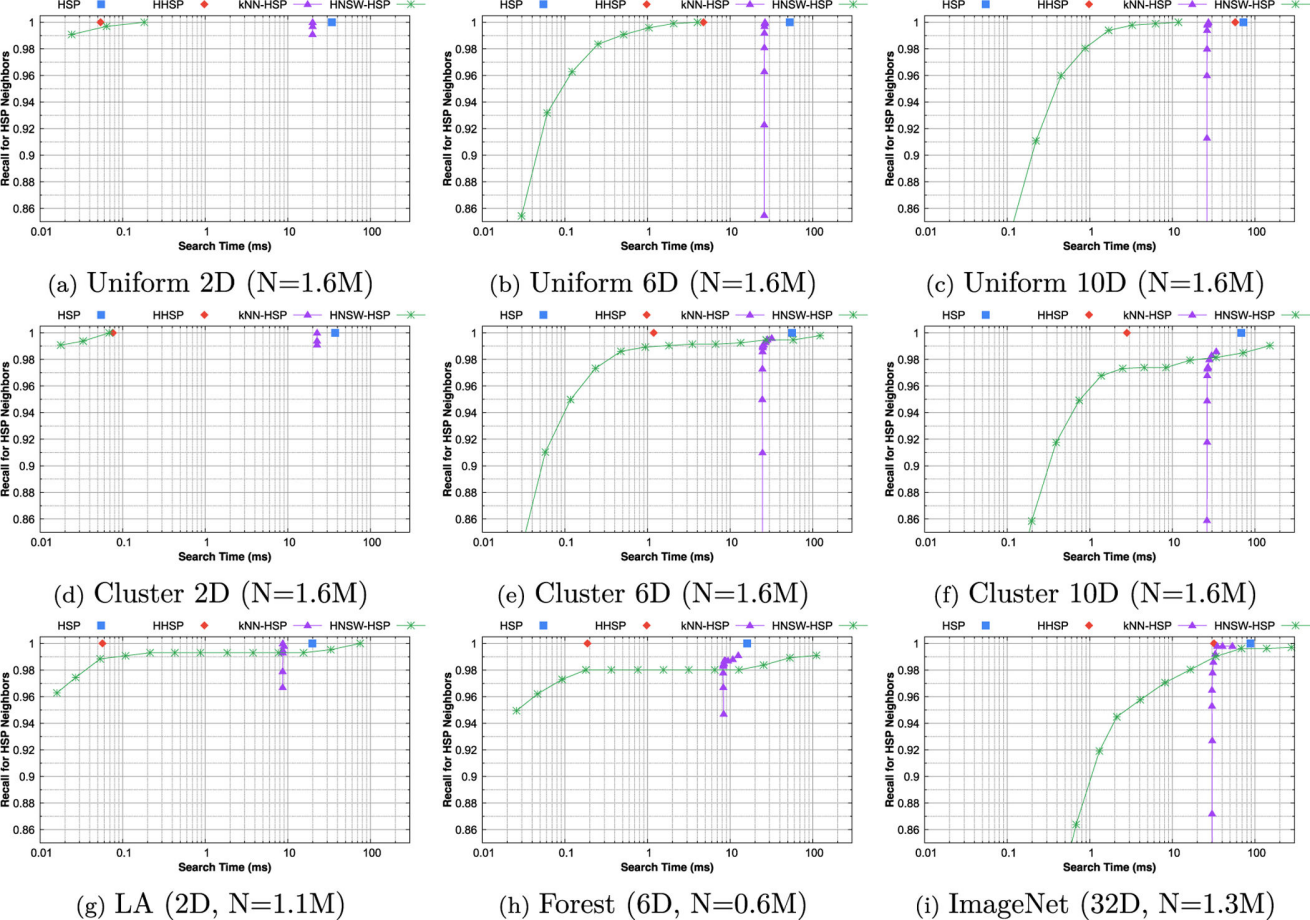
Comparing the HHSP and brute-force HSP, which produce exact results, to two approximate forms of HSP Search. The kNN-HSP performs the HSP Test on the exact kNN, while the HNSW-HSP performs the HSP Test on an approximate kNN obtained by HNSW. The time-recall curves are obtained by varying the parameter k.

The only existing scalable approach to reducing the complexity of the HSP algorithm relies on first performing an approximate kNN search to retrieve a large neighborhood around the query, and then applying the HSP Test on that neighborhood [8]. This approach leverages a state-of-the-art graph-based approximate search index, the Hierarchical Navigable Small World (HNSW) Graph [2], and this approach is denoted by HNSW-HSP. We also define a baseline for approximate HSP search, using an exact kNN search by a linear scan of the dataset, denoted by kNN-HSP. Fig. 10 shows the search time-recall curve defined by the value of k, where a large neighborhood is required to obtain high accuracy at the cost of longer search time.

Fig. 10 shows that HNSW-HSP can achieve fast search times with decent recall (90%), but high-recall (99%–100%) requires a large value of k that drastically increases the search time. In uniformly distributed data, HNSW-HSP can reach high-recall faster than the HHSP, especially in high-dimensionality, Fig. 10(a–c). However, most real datasets are not uniform, and Fig. 10(d–f) shows the HNSW-HSP struggles to reach high-recall in synthetic, clustered data, making the HHSP a better option. The results on real datasets, Fig. 10(g–i), similarly show that the HNSW-HSP struggles to reach high recall, while the kNN-HSP is actually better suited for the task. Both approximate methods are significantly out-performed by the HHSP for high-recall on the LA and Forest datasets, while the kNN-HSP and HHSP comparably outperform the HNSW-HSP on ImageNet.

### 5.7. Pivot selection

The HHSP is dependent on a hierarchical organization of the dataset. At the minimum, a two-layer hierarchy may be used, where the top layer contains a set of coarse pivots, each responsible for a set of points on the bottom layer, *i.e.*, its pivot domain, Fig. 4(a). Each member of the dataset must belong to a single pivot domain, providing minimum coverage of the dataset. This can naturally be extended to multi-layer hierarchies, where each layer of coarse-level pivots is responsible for the set of fine-level pivots on the layer below.

**Fig. 11.**
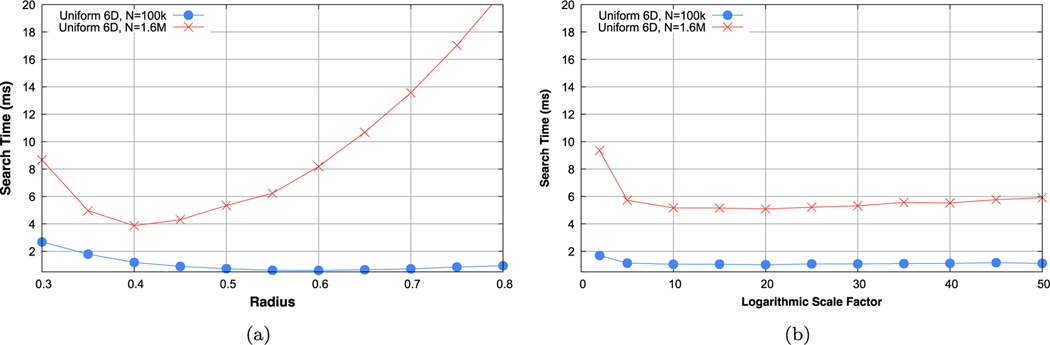
(a) Choosing the optimal radius for a 2-Layer hierarchy can be posed as a 1D optimization, and optimizing multi-layer hierarchy can be posed as a multi-dimensional optimization. (b) The scaling factor for logarithmic pivot selection can similarly follow a 1D optimization, but the result is much more stable.

This paper primarily explores a coverage-based index construction similar to that of the Cover Tree [22] with a fixed number of layers, where each layer has a predefined radius and pivots are incrementally selected to ensure coverage of the dataset. Defining an optimal hierarchy is dependent on choosing the optimal radius for each layer, which can be posed as a black-box optimization process that minimizes the number of search distances or search time on a validation set, Fig. 11(a). This optimal radius changes with the dataset dimensionality and size, and optimizing multi-layer hierarchies can be posed as a multi-dimensional optimization.

**Fig. 12.**
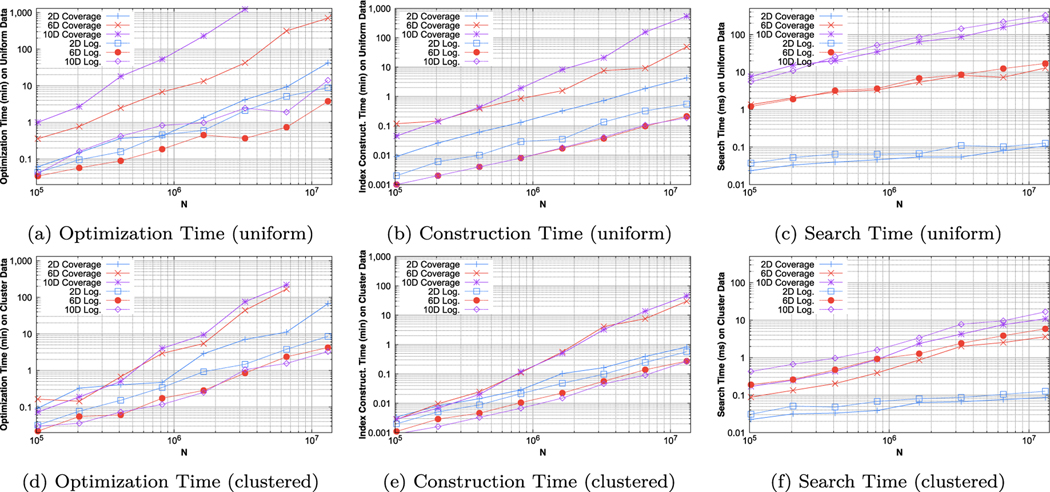
Comparing the optimized, coverage-based pivot selection to logarithmically scaling, probabilistic pivot selection for uniform data (top) and clustered data (bottom). For large dataset sizes, the optimization process can be skipped by predicting the optimal parameters.

A simpler construction process simply specifies a logarithmic scaling of pivots between layers and randomly selects pivots from the dataset to ensure that scaling, similar to that of HNSW [2]. This approach has the benefit of a single hyper-parameter, the scaling factor, which dynamically determines the number of layers. The choice of scaling factor can also be posed as a 1D optimization, Fig. 11(b). While the coverage-based pivot selection can achieve the best results, the logarithmic pivot selection can provide good results with a much lower optimization cost, Fig. 12.

## 6. Conclusions

We introduced the Hierarchical HSP (HHSP), a scalable and exact algorithm for computing Half-Space Proximal (HSP) neighbors in metric spaces with the Hilbert Exclusion property. The HHSP method employs pivot-based hierarchies and shifted generalized hyperplanes to resolve the quadratic time complexity inherent in brute-force approaches, while retaining the fundamental monotonic property of the HSP graph. The primary contributions and findings of this study are as follows:

- **Efficiency with Exactness**: HHSP achieves sublinear search complexity (Fig. 8), reducing query time by up to **348x** on real-world datasets (Table 2) while maintaining 100% recall. This eliminates the need for approximations in applications where exactness is non-negotiable, such as distance oracles and intrinsic dimensionality estimation.
- **Scalability Across Dimensions**: Experiments on synthetic data (Table 1) demonstrate that HHSP scales gracefully to high-dimensional spaces (D=10) and large datasets (N=13.1M), with search time complexity exponents α<0.6 across dimensions.
- **Robustness to Data Distribution**: HHSP outperforms approximate methods like HNSW-HSP on clustered and real-world data (Fig. 10), as its hierarchical pruning mechanism exploits local structure to retain vital long-range links (Fig. 1).
- **Practical Relevance**: The method’s efficiency on real-world datasets (e.g., *LA*, *Forest*, and *ImageNet*) underscores its applicability to domains such as geospatial analysis, bioinformatics, and multimedia retrieval.

Future work includes extending HHSP to non-Hilbert metric spaces (*e.g.*, Manhattan or Levenshtein distances), optimizing pivot hierarchies for dynamic datasets, and exploring parallel GPU/TPU implementations. Additionally, integrating HHSP into downstream tasks like graph-based machine learning or anomaly detection could unlock new use cases for exact proximity graphs.

Prior methods for HSP computation faced a trade-off between exactness and scalability. By introducing hierarchical invalidation of pivot domains, HHSP bridges this gap, enabling exact HSP neighbor retrieval at sublinear cost. This advance not only revitalizes theoretical interest in monotonic graphs but also makes them practical for modern large-scale applications. Our publicly available implementation (https://github.com/Brown-LEMS/HHSP.git) provides a foundation for further research and adoption in both academic and industrial settings.

## Figures and Tables

**Table 1:** The experimental complexity of the HHSP based on uniformly distributed data is captured by the value α for the experimental complexity $O\left(N^{\alpha }\right)$ using a least-squares fit of the function $y=\beta N^{\alpha }$.

(a) 2-Layer HHSP					

Complexity	2D	4D	6D	8D	10D

Search distances	0.505	0.493	0.503	0.546	0.616
Search time	0.673	0.616	0.663	0.685	0.757
Index construction distances	1.500	1.538	1.586	1.683	1.774
Index construction time	1.499	1.618	1.707	1.906	2.038
Index memory usage	0.801	0.845	0.850	0.842	0.714
(b) 3-Layer HHSP					

Complexity	2D	4D	6D	8D	10D

Search distances	0.377	0.326	0.357	0.464	0.586
Search time	0.521	0.434	0.424	0.635	0.680
Index construction distances	1.331	1.334	1.420	1.674	1.495
Index construction time	1.384	1.380	1.466	1.637	1.679
Index memory usage	0.811	0.856	0.887	0.889	0.891
(c) Optimal multi-layer HHSP					

Complexity	2D	4D	6D	8D	10D

Search distances	0.104	0.185	0.287	0.442	0.601
Search time	0.231	0.269	0.345	0.478	0.606
Index construction distances	1.063	1.113	1.189	1.534	1.535
Index construction time	1.194	1.196	1.294	1.621	1.763
Index memory usage	0.861	0.844	0.865	0.868	0.880

**Table 2:** Comparing the HHSP to the original HSP test on real-world datasets.

Dataset	N	Average degree	Brute force distances	Brute force time (ms)	Construction time (s)	HHSP distances	HHSP time (ms)	Time savings
LA	1,073,727	3.32	2,800,088.23	19.864	0.649	2084.59	0.057	348.49x
Forest	580,812	5.34	1,607,862.07	16.107	0.490	9192.71	0.187	86.23x
ImageNet	1,281,067	13.22	3,807,530.58	87.916	67.818	868,212.88	31.815	2.76x

## Data Availability

Data will be made available on request.
